# FAK contributes to proteinuria in hypercholesterolaemic rats and modulates podocyte F‐actin re‐organization *via* activating p38 in response to ox‐LDL

**DOI:** 10.1111/jcmm.13001

**Published:** 2016-10-05

**Authors:** Mengsi Hu, Minghua Fan, Junhui Zhen, Jiangong Lin, Qun Wang, Zhimei Lv, Rong Wang

**Affiliations:** ^1^ Department of NephrologyShandong Provincial Hospital Affiliated to Shandong UniversityJinanChina; ^2^ Department of Obstetrics and GynecologyThe Second Hospital of Shandong UniversityJinanChina; ^3^ Department of PathologySchool of MedicineShandong UniversityJinanChina

**Keywords:** podocyte, hypercholesterolaemia, ox‐LDL, FAK, p38, cytoskeleton

## Abstract

Focal adhesion kinase (FAK) is a non‐receptor protein tyrosine kinase that regulates cell adhesion, proliferation and differentiation. In the present study, a rat model of high fat diet‐induced hypercholesterolaemia was established to investigate the involvement of FAK in lipid disorder‐related kidney diseases. We showed focal fusion of podocyte foot process that occurred at as early as 4 weeks in rats consuming high fat diet, preceding the onset of proteinuria when aberrant phosphorylation of FAK was found. These abnormalities were ameliorated by dietary intervention of TAE226, a reported inhibitor of FAK. FAK is also an adaptor protein initiating cascades of intracellular signals including c‐Src, Rho GTPase and mitogen‐activated protein kinase (MAPK). P38 MAPK belongs to the latter and is centrally involved in kidney diseases. Our cell culture data revealed oxidized low‐density lipoprotein (ox‐LDL) triggered hyper‐phosphorylation of FAK and p38, ectopic expression of cellular markers (manifested as decreased WT1, podocin and NEPH1, and increased vimentin and mmp9), and re‐arrangement of F‐actin filaments with enhanced cell motility; these mutations were significantly rectified by FAK shRNA. Notably, pre‐treatment of p38 inhibitor did not alter FAK activation, albeit its deletion of p38 hyper‐activity and attenuation of cellular abnormalities, demonstrating that p38 acted as a downstream effector of FAK signalling and ox‐LDL damaged podocytes in a FAK/p38‐dependent manner. This was further identified by animal data that p38 activation was also abrogated by TAE226 treatment in hypercholesterolaemic rats, suggesting that FAK/p38 axis might also be involved in *in vivo* events. These findings provided a potential early mechanism of hypercholesterolaemia‐related podocyte damage and proteinuria.

## Introduction

Lipid metabolism disorders, hypercholesterolaemia in particular plays a critical role in the development of glomerular diseases 1. Podocytes are a crucial component of kidney filtration barrier and their dysfunction plays a fundamental role in a variety of proteinuric kidney disorders 2. They are directly exposed to lipoproteins in pathological states like in nephrotic syndrome and can uptake lipids *via* its scavenger receptors 3, 4. Evidence has shown that hypercholesterolaemia at early stage may aggravate renal injury primarily *via* podocytes rather than other residential cells and forced podocyte injury could secondarily lead to mesangial sclerosis 5, 6, 7. Therefore, further understanding of mechanisms of hypercholesterolaemia‐associated podocyte injury is of great importance.

Focal adhesion kinase (FAK) is a 125‐kD non‐receptor protein tyrosine kinase, and functions in regulating cell adhesion, proliferation and differentiation in a variety of cells types 8. Hyper‐phosphorylation of FAK was found in glomerular diseases such as lupus nephritis and anti‐glomerular basement membrane (anti‐GBM) disease 9, 10. In diabetic rats, boosted tyrosine phosphorylation of FAK was present in renal glomeruli, with unchanged total FAK expression and unregulated integrin β1 expression 11. Notably, CD36‐dependent sustained activation of FAK was revealed in ox‐LDL‐stimulated mouse macrophages *in vitro*, suggesting that FAK was a potential participant in lipid metabolism 12. Prior studies also demonstrated that FAK modulated cell motility by influencing the cytoskeleton, structures of cell adhesion sites and membrane protrusions 13. Activated by numerous stimuli, FAK functions as a bidirectional linkage between the actin cytoskeleton and the cell‐matrix interface, *via* its signals to downstream effectors such as paxillin 14, which was involved in high glucose‐induced podocyte cytoskeletal re‐arrangement *in vitro* 15. Phosphoinositide 3‐kinase (PI3K), another important substrate of FAK signalling, was reported to contribute to abnormal podocyte actin cytoskeleton and thereafter proteinuria *via* its interactions with nephrin 14, 16. Therefore, investigations of FAK in lipid disorder‐related kidney damage and its involvement in podocyte cytoskeletal dynamics are of intense interest.

Focal adhesion kinase is also a large adaptor protein that initiates a cascade of intracellular signals in response to adhesion, including c‐Src, Rho GTPase and mitogen‐activated protein kinase (MAPK) activation 17, 18. P38 MAPK (p38) is a member of the MAPK family and is centrally involved in diverse cellular processes, including inflammation, cell differentiation, cell growth and cell death 19. A wealth of data has indicated that p38 is also a key regulator in multiple renal diseases 20. In diabetic nephropathy, p38 was phosphorylated in renal proximal tubular epithelial cells (PTEC) and involved in epithelial‐mesenchymal transition of PTECs, which has been viewed as a principal source of fibroblasts in renal fibrosis 21, 22. It contributed to ox‐LDL‐induced macrophage proliferation *via* a granulocyte/macrophage colony‐stimulating factor‐dependent manner, which could be suppressed by statins 23. The cross‐talk between MAPK and scavenger receptors has been well‐elaborated by later reports that p38 was responsible for the foam cell formation by promoting overexpression of CD36, a scavenger receptor that accounts for the uptake of ox‐LDL in mesangial cells 24, 25. However, data on the role of p38 in lipid disorder‐related podocyte damage and its potential interplay with FAK are largely unknown.

In the present study, we showed early onset of podocyte foot process effacement and proteinuria in a rat model of hypercholesterolaemia, with aberrant phosphorylation of FAK and p38, which were restored and prohibited by oral administration of TAE226, a reported inhibitor of FAK. Our cell culture data further revealed that a re‐organization of F‐actin filaments and promoted cell migration were driven by ox‐LDL stimulation in a FAK/p38 signalling‐dependent manner; blockade of this signalling pathway significantly reinstated ox‐LDL‐forced F‐actin cytoskeleton re‐assembly and rectified podocyte hyper‐motility. These findings provided evidence for a potential early mechanism of hypercholesterolaemia‐associated podocyte damage and proteinuria.

## Materials and methods

### Reagents

Antibodies specific for podocin, synaptopodin, vimentin, mmp9, WT1 and phospho‐FAK (phospho Y397) were from Abcam (Cambridge, MA, USA). Anti‐NEPH1 antibodies purchased from Santa Cruz Biotechnology, Inc. (Santa Cruz, CA, USA). Anti‐p38 MAPK, phospho‐p38 MAPK (Thr180/Tyr182) and FAK antibodies were purchased from Cell Signaling Technology, Inc. (Danvers, MA, USA). Anti‐β‐actin antibodies were obtained from Proteintech (Chicago, IL, USA). Secondary antibodies were from Jackson ImmunoResearch Laboratories, Inc. (West Grove, PA, USA) and Dylight 594 conjugated secondary antibodies were from Pierce, Thermo Fisher Scientific Inc. (Rockford, IL, USA). Mmp9 ELISA kits were from Abcam. The p38 inhibitor SB203580 was from Selleck (Houston, TX, USA). LDL and FITC‐Phalloidin were purchased from Sigma Chemical Company (St Louis, MO, USA). The FAK inhibitor TAE226 was from Novartis Pharmaceuticals (Plantation, FL, USA). For *in vitro* use, SB203580 was first diluted in DMSO as a stock liquid (50 mM); for further use, the stock liquid was added for a terminal concentration at 5 μg/ml 26. For oxidation, LDL (5 mg/ml) were mixed with 5 μmol CuSO_4_, incubated for 18 hrs at 37°C, and oxidation was evaluated as described previously 27.

### Animal studies

80 male Wistar rats, weighing 200–220 g, were purchased from the Animal Center affiliated to Shandong University. All animal studies were carried out with the review and approval of the animal care and use committee of Shandong University. Rats were randomly allocated into four groups, either fed with a normal chow (NC) or a 6 weeks high cholesterol diet (HC) (2% cholesterol, 1% cholic acid and 10% lard diet) with or without dietary intervention 28. For rats consuming high fat diet, TAE226 (25 mg/kg) or methylcellulose as a vehicle was orally administered as dietary intervention once a day until rats were killed 29, 30. At the end of 0, 2, 4 and 6 weeks, blood samples were taken from the tail vein and stored at −80°C until further determination of plasma creatinine, cholesterol, LDL, HDL and triglycerides using Keygen's reagents (Nanjing, China); urine was collected for the determination of urinary protein when the rats were weighed and placed in metabolic cages for 24 hrs, with free access to food and water. At the end of 4 and 6 weeks, rats were killed and the kidneys were harvested for the following experiments.

### Cell culture

Conditionally immortalized mouse podocytes were kindly provided by Professor Peter Mundel (Massachusetts General Hospital, Boston, MA, USA) *via* Professor Jie Ding (Peking University). In brief, podocytes were cultured on type I collagen in RPMI 1640 supplemented with 10% foetal bovine serum (Life Technologies Corporation, Calsbad, CA, USA), 100 U/ml penicillin, and 100 mg/ml streptomycin under permissive conditions 33°C plus 10 U/ml mouse recombinant γ‐interferon (Pepro Technology, Rocky Hill, NJ, USA). Cell differentiation was induced by maintaining podocytes on type I collagen at 37°C without γ‐interferon under non‐permissive conditions for at least 14 days 31, 32. To determine the effects of ox‐LDL on podocytes, ox‐LDL was added (20 μg/ml) to the culture medium for 24 hrs. For p38 inhibition, cells were pre‐treated with SB203580 (terminal concentration at 5 μg/ml) for 1 hr before stimulations.

### RNA interference

Plasmid vectors that target FAK (5′‐CCTGGCATCTTTGATATTATA‐3′) (FAK shRNA) and a negative control sequence (5′‐GACTTCATAAGGCGCATGC‐3′) (scrambled shRNA) were purchased from Cyagen Biosciences Inc. (Guangzhou, China). Transfection of podocytes was performed (1 × 10^6^ cells/well in a 6‐well plate) with the indicated plasmids using Lipofectamine2000 reagent (Invitrogen, Life Technologies Corporation). Western blot analysis and real‐time reverse transcriptase PCR were used to validate the efficiency of FAK gene knockdown. After a 24‐hr incubation with FAK‐shRNA or scrambled shRNA, podocytes were then either treated with or without ox‐LDL (20 μg/ml) for an additional 24 hrs. Cells that were not transfected and incubated with normal culture medium for 24 hrs were the controls.

### Flow cytometry

Apoptosis was assessed using FITC Annexin V Apoptosis Detection Kit from BD Biosciences (Franklin Lakes, NJ, USA). The staining protocol was performed according to the manufacturer's instructions. Briefly, cells with or without treatment of ox‐LDL for 24 hrs were trypsinized and collected by centrifugation at 500 × g for 5 min. at room temperature. After washed twice with cold PBS, cells were re‐suspended in 1× binding buffer at concentration of about 1 × 10^6^ cells/ml. Then 100 μl of the solution (1 × 10^5^ cells) was transferred to a 5 ml culture tube, in which 5 μl Annexin V and propidium iodide (PI) were added. Cells were gently mixed and incubated at room temperature in the dark for 15 min. Next 400 μl of 1× binding buffer was added to each tube. And the cells were detected using FACSVantage SETM flow cytometry (BD Biosciences) within 1 hr. Cells that were Annexin V positive and PI negative were considered apoptotic.

### SDS‐PAGE and western blot analysis

The total protein was extracted from rat kidney cortices or podocytes under different conditions with ice‐cold lysis buffer containing proteinase inhibitors and phosphatase inhibitors. 50 μg of total protein was separated by SDS‐PAGE and transferred to polyvinylidene fluoride membranes; the membranes were then blocked with 5% milk or bovine serum albumin (BSA) for 1 hr and incubated at 4°C overnight with primary antibodies against the following target proteins: FAK (1:1000), phospho‐FAK (1:1000), phos‐p38 MAPK (1:1000), p38 MAPK (1:1000), WT1 (1:2000), NEPH1 (1:200), podocin (1: 1000), vimentin (1:2000), synaptopodin (1:1000), mmp9 (1:1000) and β‐actin (1:1000). The membranes were then washed three times with PBST for 10 min. and incubated with species‐specific peroxidase‐conjugated secondary antibodies diluted in blocking buffer for 2 hrs at room temperature. Specific bands were detected using the ECL system and the Bio‐Rad electrophoresis image analyser (Bio‐Rad, Hercules, CA, USA).

### RNA extraction and real‐time reverse transcriptase PCR

Real‐time reverse transcriptase PCR was used to detect the gene expression in cultured podocytes and rat kidney cortices. Extraction and concentration calculation of total RNA were described as previously 32. Aliquots of total RNA (1.0 μg each) from each sample were reverse transcribed into cDNA according to the instructions of PrimeScript^®^ RT Reagent Kit (Takara, Dalian, China). Briefly, after reverse transcription of total RNA, cDNA was used as a template for the PCR reactions using gene‐specific primer pairs. Amplification was done using SYBR^®^ Premix Ex Taq^™^ Kit (Takara) in the LightCycler^®^ 480 Real‐Time PCR system (Roche Applied Science, F. Hoffmann‐La Roche Ltd, Pleasanton, CA, USA). The primers were purchased from Sangon Biotech Co., Ltd (Shanghai, China). The sequences were designed as follows (Table 1).

**Table 1:** Primers for immortalized mouse podocytes and rat kidneys

Gene	Primer	Sequence
Wt1		
Mouse	Forward	5′‐CATACCCAGGCTGCAATAAGA‐3′
	Reverse	5′‐CCTGTGTGTCTCCTTTGGTGT‐3′
Rat	Forward	5′‐GTGCTCTGCCCACTCTTGA‐3′
	Reverse	5′‐TGGAACAACCGCTCTAATCC‐3′
Kirrel		
Mouse	Forward	5′‐GCATCCTGGTCGTCTTCTCT‐3′
	Reverse	5′‐ATTCACCGTCTCCACCTTGA‐3′
Rat	Forward	5′‐GCGGTAAGGTGGAGTGCTT‐3′
	Reverse	5′‐TGATGGTGAGAGTGGACAGC‐3′
Mmp9		
Mouse	Forward	5′‐CCTGTGTGTTCCCGTTCAT‐3′
	Reverse	5′‐CCTGGTCATAGTTGGCTGTG‐3′
Rat	Forward	5′‐GCCGACTTATGTGGTCTTCC‐3′
	Reverse	5′‐TGCCCGAGTGTAACCATAGC‐3′
Vim		
Mouse	Forward	5′‐AGATGCGTGAGATGGAAGAGA‐3′
	Reverse	5′‐GTTCAGGGAAGAAAAGGTTGG‐3′
Rat	Forward	5′‐TCCGCCAGCAGTATGAAAGT‐3′
	Reverse	5′‐TCCGGTATTCGTTTGACTCC‐3′
Actb		
Mouse	Forward	5′‐ AAGACGAGGAGGAACTGAAC ‐3′
	Reverse	5′‐ CAAATCGGA CAACAAGACG ‐3′
Rat	Forward	5′‐CCCATCTATGAGGGTTACGC‐3′
	Reverse	5′‐TTTAATGTCACGCACGATTTC‐3′
Synpo		
Mouse	Forward	5′‐CGGAGAATCAAAACCCTCAG‐3′
	Reverse	5′‐CAGGACACTGCCATCAGACT‐3′
Ptk2		
Mouse	Forward	5′‐GACCCCAGAAAGAAGGTGAAC‐3′
	Reverse	5′‐TCTCCAATACAGCGTCCAAGT‐3′
Nphs2		
Rat	Forward	5′‐TGCCTGGACACCTATCACAAG‐3′
	Reverse	5′‐AACTGGATGGCTTTGGACAC‐3′

### Immunofluorescence

The rat kidneys were paraffin embedded, sectioned at 4 μm, and subjected to antigen retrieval followed by blocking with 1% BSA for 1 hr at room temperature to block non‐specific binding. Cultured podocytes under different conditions were plated onto different 6‐well plates and fixed in 4% paraformaldehyde for 10 min., followed by 1% BSA (with 0.3% Triton X‐100) for 1 hr at room temperature to block non‐specific binding. Immunostaining was performed with appropriate primary antibodies at 4°C overnight and Dylight 594‐conjugated IgG at room temperature for 1 hr for visualization. 4′,6‐diamidino‐2‐phenylindole (DAPI) was used to visualize the nuclei. For the staining of F‐actin, FITC‐phalloidin (50 μg/ml) was used before DAPI at room temperature for 1 hr. Images were observed and captured on an inverted phase/fluorescence microscope (Leica Microsystems GmbH, Wetzlar, Germany).

### ELISA

ELISA was used to detect concentrations of mmp9 in the supernatants of different cell cultures, according to the manufacturer's instructions.

### Transmission electron microscopy

For transmission electron microscopy (TEM) analysis, small pieces of rat renal cortex were fixed in 2% glutaraldehyde and 1% osmic acid for 2 hrs at 4°C. After two washes in PBS, the samples were dehydrated with an ethanol gradient, washed twice with propylene oxide, soaked in ethoxyline resin overnight, and mounted at 60°C for 48 hrs. Ultrathin sections (70 nm+) were cut with an ultramicrotome and then viewed under H‐7500 transmission electron microscope (Hitachi, Tokyo, Japan). Images with a final magnification of approximately 10,000× were obtained.

### Scratch‐wound assay

Confluent monolayers of podocytes seeded onto type I collagen‐coated six‐well plates were pre‐treated with different culture conditions and scraped with a 10 μl pipette and then allowed to migrate for 12 hrs. The percentage of wound closure was captured using an inverted phase contrast microscope (Nikon, Tokyo, Japan) and calculated using NIH ImageJ. Migratory rates were calculated as (A − B)/A × 100%, where A and B reflect the width of the wound at 0 or 12 hrs respectively. The data denote the mean ± S.D. of 6 independent experiments.

### Transwell migration assay

Transwell cell‐culture inserts (pore size 8 μm; Corning Costar Corp., Cambridge, MA, USA) were rinsed once with PBS and placed in RPMI1640 with 10% foetal bovine serum in the lower compartment. The heights of the medium in the upper and lower compartments were maintained at similar levels; thus, bulk flow was not because of a hydrostatic pressure gradient. Podocytes pre‐treated with different conditions were harvested with trypsin and re‐suspended in serum‐free RPMI1640 medium. The upper chambers were seeded with 1 × 10^4^/ml cells, which were then allowed to attach at 37°C for 6 or 12 hrs, respectively. Next, non‐migratory cells were removed from the upper surface of the membrane, and migrated cells were fixed with 4% paraformaldehyde and stained with haematoxylin. The number of migrated cells in the centre of a membrane (one field) was counted using phase contrast microscopy (Leica Microsystems GmbH). The data presented denote the mean ± S.D. of 6 independent experiments.

### Statistical analyses

Experiments were performed at least three times. Values were reported as mean ± S.D. Data were analysed using SPSS 19.0 software (IBM, Armonk, NY, USA). Statistical significance was assessed using one‐way anova and LSD *t*‐test and *P* < 0.05 were considered to be statistically significant.

## Results

### Dietary intervention of TAE226 alleviated hypercholesterolaemia‐associated proteinuria

**Figure 1:**
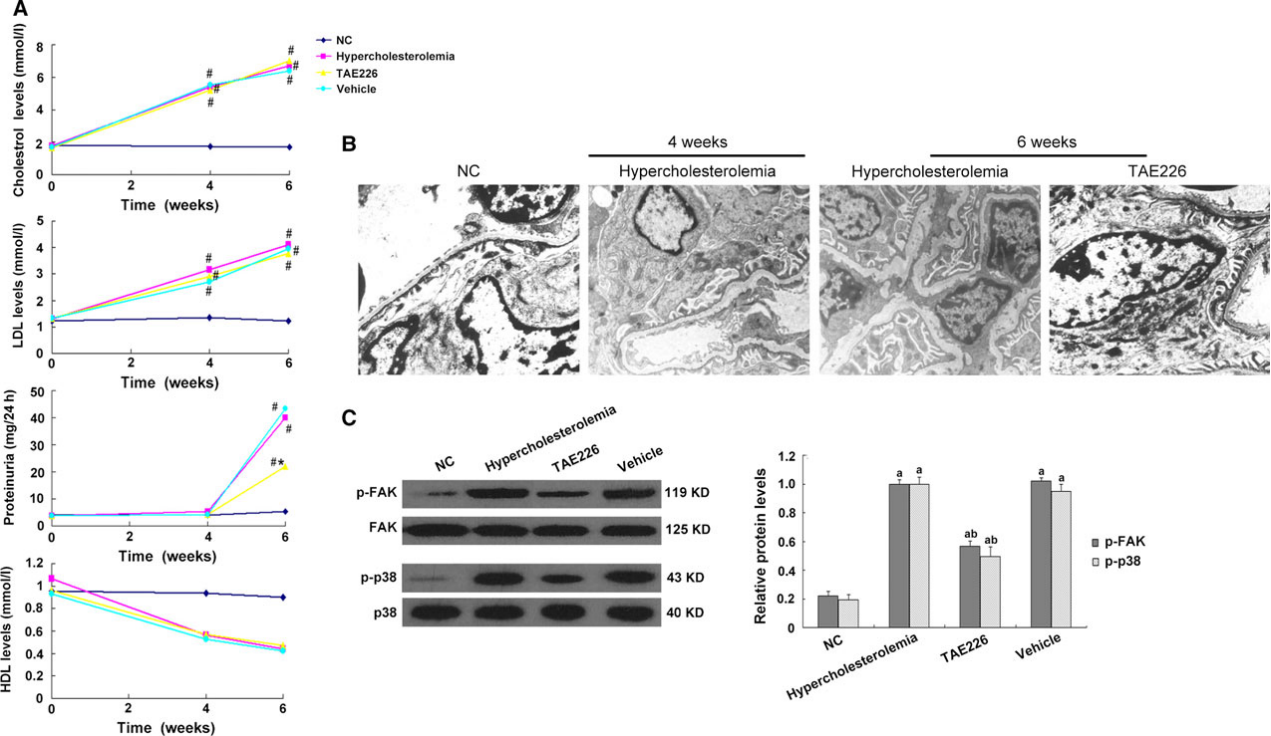
Hypercholesterolaemia damaged glomerular podocytes and stimulated the activation of FAK and p38. (**A**) High fat diet elevated plasma levels of cholesterol and LDL, and decreased HDL levels in a time‐dependent manner in comparison with NC rats. Hypercholesterolaemia induced the onset of proteinuria in rats at 6 weeks, which was ameliorated by TAE226 treatment. Nevertheless, TAE226 intervention showed no remarkable effects on lipids levels. Values denote the mean ± S.D.; ^#^ *P* < 0.05 *versus* NC and *P < 0.05 *versus* hypercholesterolaemic rats. (**B**) By TEM, we observed that at as early as 4 weeks, focal fusion of podocyte foot process occurred in rats on a high‐fat diet, which developed to diffused effacement at 6 weeks with normal cytoplasmic organoids; these sub‐cellular changes were rectified by TAE226, manifested as partially reinstated podocyte foot process effacement, with slightly irregularly thickened GBM, magnification 10,000×. (**C**) Western blot showed FAK and p38 were activated by 6 weeks of high fat diet and were both abrogated by TAE226 administration. Rats fed with normal chow or vehicle were the controls. Values denote the mean ± S.D.; ^a^ *P* < 0.05 *versus* NC and ^b^ *P* < 0.05 *versus* hypercholesterolaemic rats.

In comparison with NC rats, high fat diet caused notable increase in cholesterol and LDL levels, but significant decrease in HDL levels at 4 and 6 weeks (*P* < 0.05; Fig. 1A), without affecting other biochemical parameters (Table 2). These rats developed mild proteinuria at 6 weeks, which was markedly reduced by oral administration of FAK inhibitor TAE226 (*P* < 0.05; Fig. 1A). However, it is noteworthy that TAE226 intervention did not significantly alter the circulating levels of cholesterol, LDL or HDL (Fig. 1A), suggesting that TAE226 might interfere with early proteinuria *via* non‐lipid‐lowering mechanisms.

**Table 2:** Metabolic data of four groups at 6 weeks of experiment

Groups	Normal diet	High fat diet	TAE226 iintervention	Vehicle
Body weight (g)	364.61 ± 36.23	376.31 ± 39.29	371.81 ± 25.91	374.81 ± 27.46
Blood Pressure (mmHg)	126 ± 2	129 ± 1	127 ± 2	126 ± 1
Plasma creatine (mM)	58.29 ± 6.13	59.71 ± 2.27	59.34 ± 6.13	65.56 ± 4.56
Plasma triglycerides (mM)	0.71 ± 0.01	0.73 ± 0.01	0.70 ± 0.01	0.73 ± 0.01

Data are mean ± S.D.; *n* = 20 in each group.

### Inhibition of FAK ameliorated the damage of glomerular podocytes in hypercholesterolaemic rats

**Figure 2:**
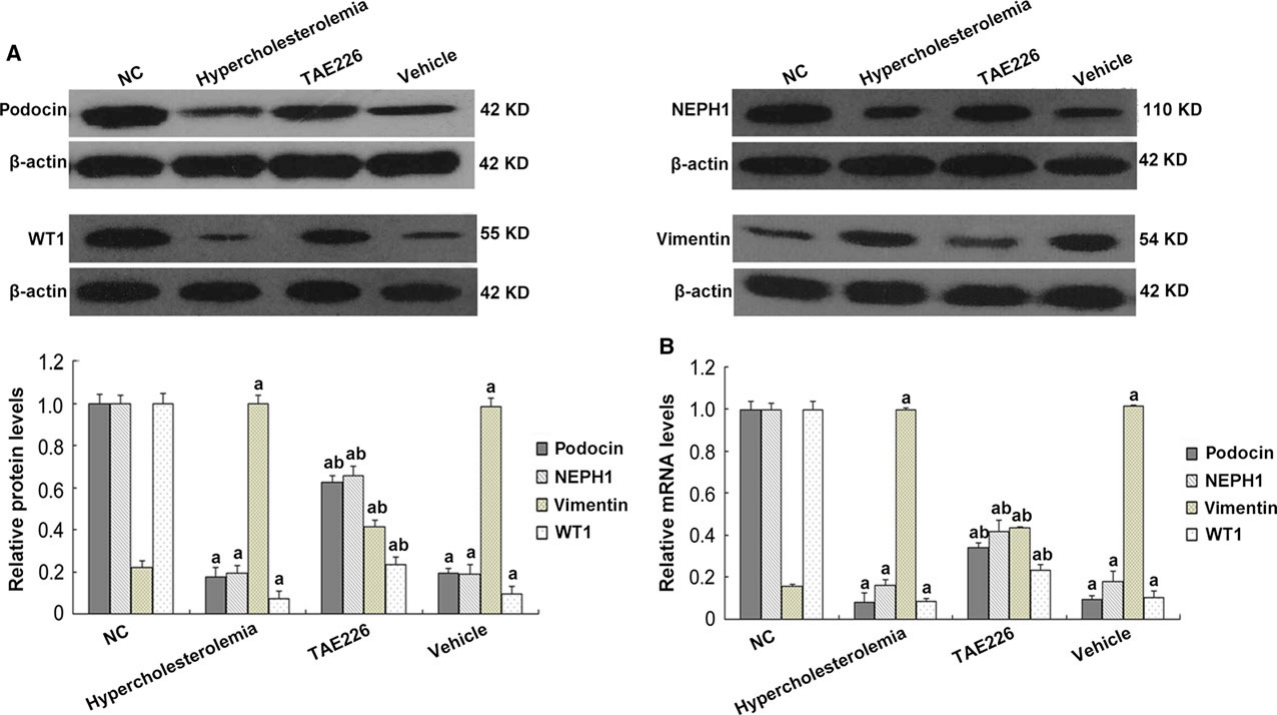
Determination of cellular and injury markers in rat glomerular podocytes by western blot and real‐time PCR. Hypercholesterolaemia decreased the expression of podocyte markers podocin, WT1 and NEPH1, and promoted vimentin expression on both protein (**A**) and mRNA levels (**B**); these abnormalities were partially reversed by TAE226. Rats fed with normal chow or vehicle were the controls. Values denote the mean ± S.D.; ^a^ *P* < 0.05 *versus* NC and ^b^ *P* < 0.05 *versus* hypercholesterolaemic rats.

**Figure 3:**
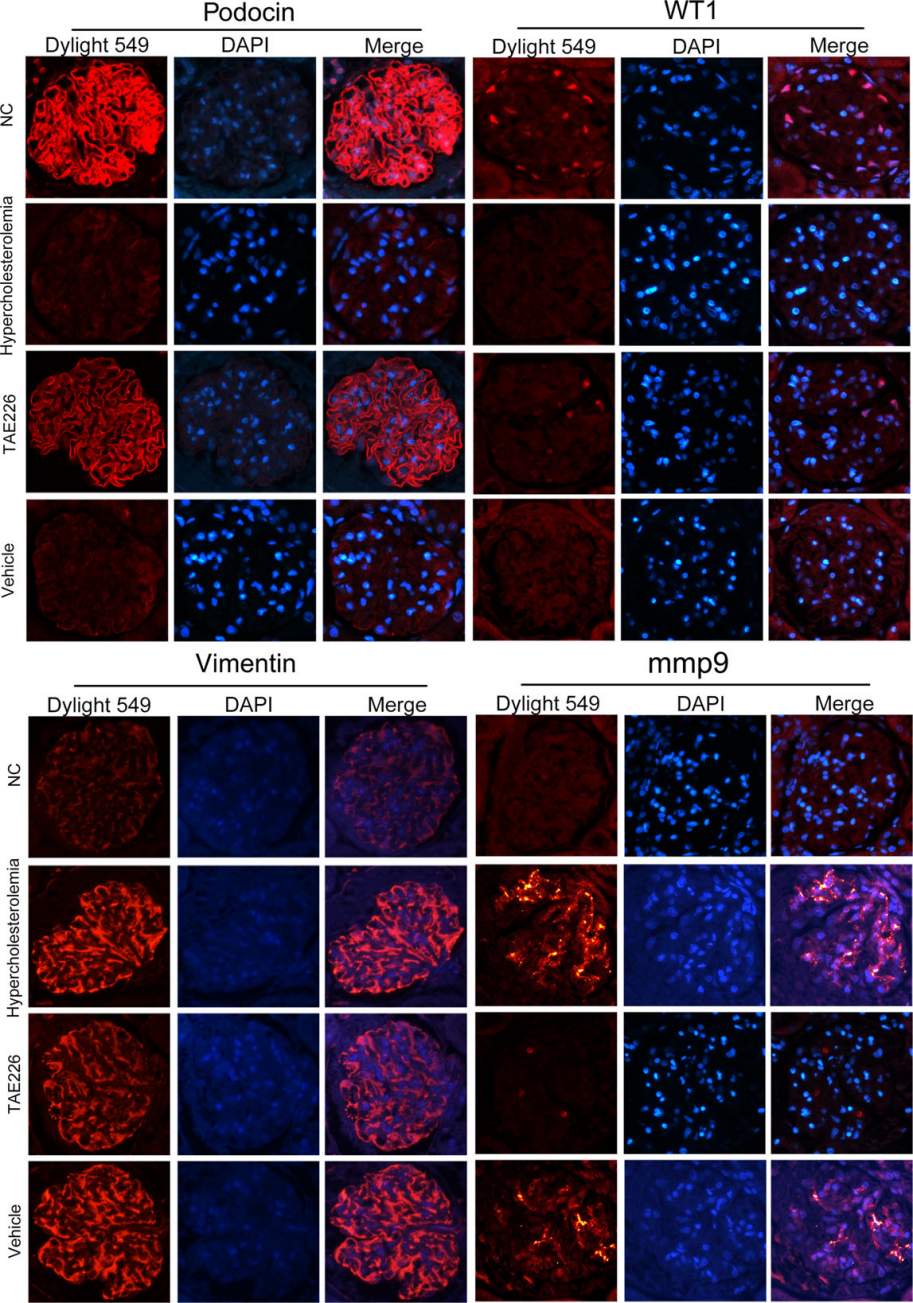
Alterations of cellular and injury markers in glomerular podocytes by Immunofluorescence. In NC rats, podocyte specific markers were highly expressed. WT1 was abundant in the podocyte nuclei; podocin was widely distributed in the cytoplasm that formed a linear pattern around the glomeruli. In HC rats, immunostaining of these molecules were markedly reduced: WT1 staining was weakened and barely seen in the nucleui with podocin scattered in a faint and intermittent linear pattern. Hypercholesterolaemia also resulted in increased staining of injury markers vimentin and mmp9 in comparison with their counterparts, in which mmp9 staining was undetectable and vimentin expressed in cytoplasm at a relatively low level. Inhibition of FAK activation by TAE226 partially reversed these mutations, as enhanced staining of WT1 and podocin and decreased staining of vimentin and mmp9 were observed, compared with untreated HC rats, magnification 400×.

Podocyte dysfunction was closely correlated with protein leakage 33, 34. Using TEM, we observed that at as early as 4 weeks, focal podocyte foot process fusion occurred in rats consuming high‐fat diet, which developed to diffused effacement but normal cytoplasmic organoids at week 6, accompanied by mild proteinuria (Fig. 1B), indicating that hypercholesterolaemia‐associated proteinuria was preceded by early podocyte lesion. Previous studies have illustrated that FAK was involved in kidney diseases and also a potential participant in lipid metabolism 12. Therefore, we were interested to explore the alterations of FAK expression in HC rats. Compared with NC rats, hyper‐phosphorylation of FAK was found in the renal cortex of rats treated with high fat diet at 6 weeks (*P* < 0.05; Fig. 1C), with enhanced expression of vimentin and matrix metalloproteinase9 (mmp9), and markedly decreased podocyte specific markers WT1, podocin and NEPH1 (*P* < 0.05; Figs 2 and 3). WT1 protein is critical to the maintenance of the differentiated features of adult podocytes, mutations of which account for podocyte dysfunction 35. NEPH1 and podocin localize to the slit diaphragm (SD) of podocytes and interact with the trans‐membrane adhesion protein nephrin; mutations of these proteins are observed in patients with inherited and sporadic nephrotic syndromes 36. Vimentin is one of the intermediate filament proteins, which is expressed at a basic level but markedly up‐regulated in podocytes of diseased kidneys 37. Mmp9 is one of the zinc‐containing endopeptidases involved in remodelling the extracellular matrix (ECM), and its aberrant activation is correlated with kidney fibrosis 38.

Since TAE226 showed a protective effect on early proteinuria, we further determined whether it could benefit glomerular podocytes. Results revealed that TAE226 substantially reduced FAK over‐activation compared with untreated HC rats (*P* < 0.05; Fig. 1C). Ultrastructural mutations on TEM were also rectified by TAE226 treatment, manifested as partially reinstated podocyte foot process effacement, with slightly irregularly thickened GBM (Fig. 1B). Similar amelioration of cellular markers were also observed in TAE226‐treated rats (*P* < 0.05; Fig. 2), which were further identified by immunofluorescence (Fig. 3). In NC rats, WT1 was abundant in the podocyte nuclei but was weakened and barely seen in HC rats. Hypercholesterolaemia also caused altered staining of podocin, from a clear linear pattern around the glomeruli to a faint and intermittent staining. Concomitantly, substantially increased staining of vimentin and mmp9 was revealed in HC rats in comparison with their controls in which mmp9 was undetectable and vimentin expressed in cytoplasm at a relatively low level. These abnormalities were essentially prevented by oral administration of TAE226, indicating that inhibition of FAK markedly restored the damage of glomerular podocytes.

### Ox‐LDL treatment induced podocyte impairment through activating FAK signalling *in vitro*

**Figure 4:**
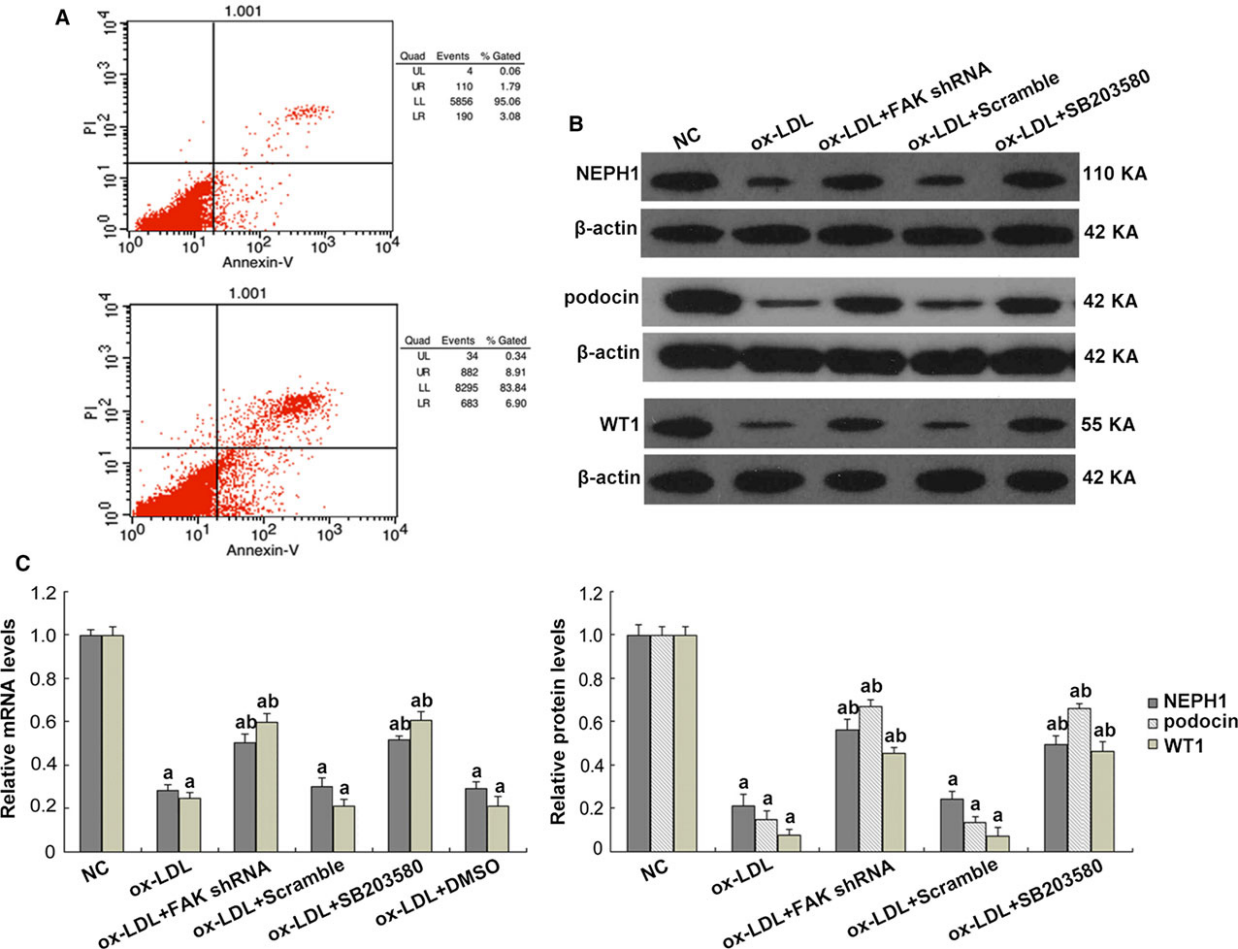
Ox‐LDL induced decreases in cellular markers in cultured mouse podocytes. (**A**) Flow cytometry showed a great proportion of podocytes were viable after a 24 hrs of ox‐LDL treatment. (**B** and **C**) Western blot revealed that substantial reductions in NEPH1 and WT1 expression were induced by ox‐LDL. FAK gene knockdown or pre‐treatment of p38 inhibitor SB203580 significantly preserved the expression of these markers on both protein and mRNA levels. Ox‐LDL also resulted in suppressed expression of podocin by western blot. Podocytes under normal conditions or transfected with scrambled shRNA were the controls. Values denote the mean ± S.D.; ^a^ *P* < 0.05 *versus* NC and ^b^ *P* < 0.05 *versus* ox‐LDL treatment.

**Figure 5:**
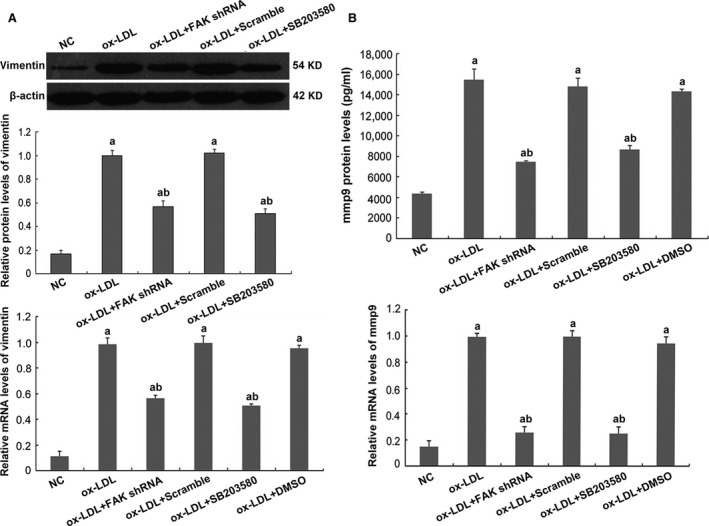
Elevated expression of injury markers triggered by ox‐LDL in cultured mouse podocytes. (**A**) Western blot and real‐time PCR presented that ox‐LDL led to enhanced vimentin expression on both protein and mRNA levels. (**B**) ELISA showed that excessive expression and secretion of mmp9 were induced by ox‐LDL; aberrant expressions of these two proteins were abolished by FAK shRNA or pre‐treatment of SB203580. Podocytes under normal conditions or transfected with scrambled shRNA were the controls. Values denote the mean ± S.D.; ^a^ *P* < 0.05 *versus* NC and ^b^ *P* < 0.05 *versus* ox‐LDL treatment.

**Figure 6:**
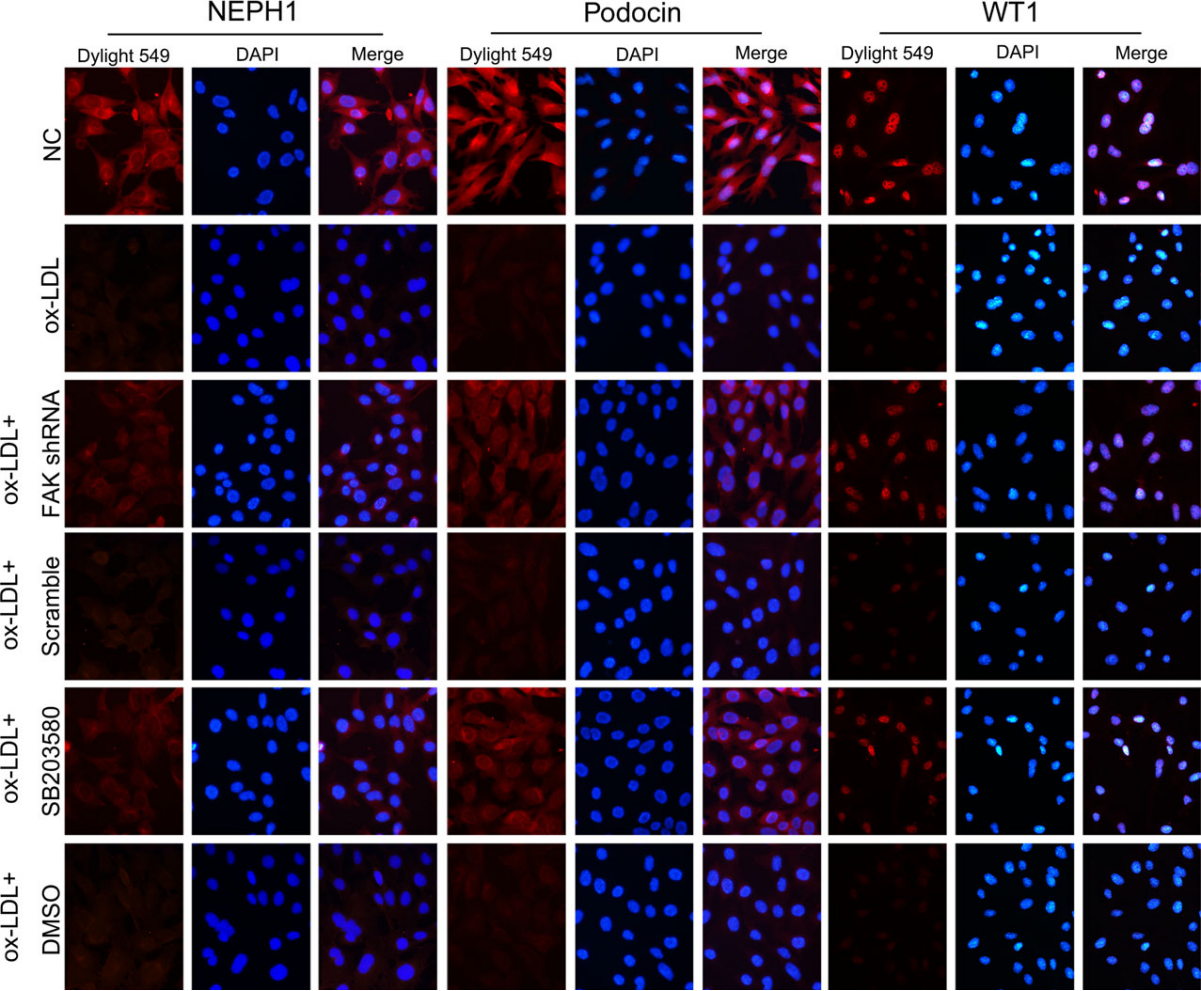
Alterations of specific markers in cultured podocytes by immunofluorescence. Immunofluorescence indicated that under normal conditions, podocyte specific markers were strongly stained, with WT1 centred in the cell nuclei and NEPH1 and podocin distributed in the cytoplasm. Ox‐LDL stimulation for 24 hrs weakened the staining of these specific markers. FAK shRNA transfection or the addition of p38 inhibitor showed an amelioration of these proteins, demonstrated by enhanced staining of WT1, NEPH1 and podocin, compared with podocytes without transfection or transfected with scrambled shRNA, magnification 400×.

**Figure 7:**
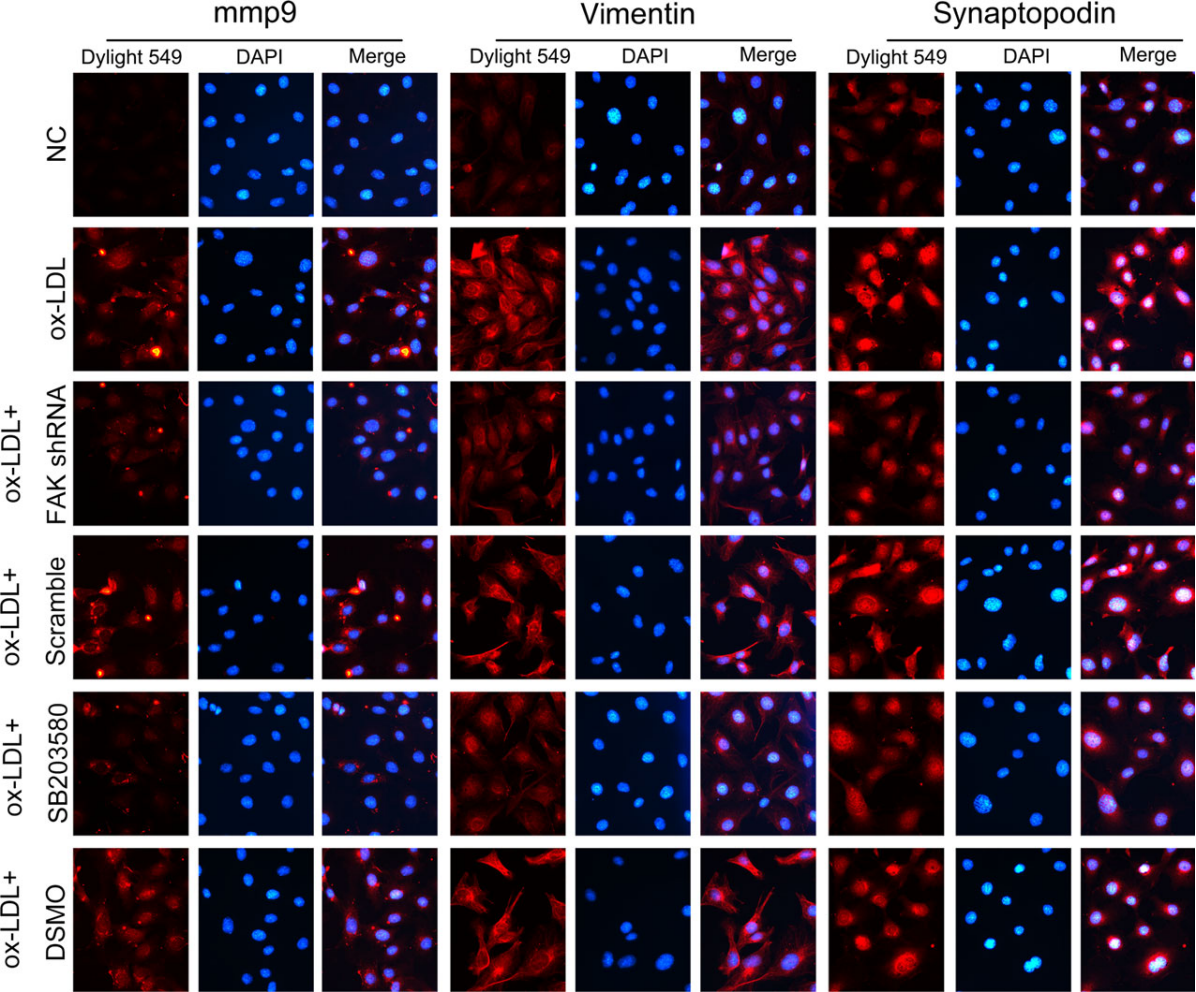
Alterations of injury markers and synaptopodin expression in cultured podocytes by immunofluorescence. Immunofluorescence suggested that under normal conditions, mmp9 was weakly stained within the cell but scarcely secreted, and vimentin was moderately expressed in the podocyte cytoplasm; promoted expression and secretion of mmp9 that was seen at the inter‐surface of the cells and enhanced staining of vimentin was seen in podocytes after ox‐LDL treatment for 24 hrs. Forced staining of vimentin and mmp9 was markedly reduced by FAK shRNA or pre‐treatment of SB203580. Furthermore, we showed that synaptopodin expression were up‐modulated by ox‐LDL treatment for 24 hrs, compared with podocytes under normal culture medium, which were partially reinstated by FAK gene knockdown or p38 activation abrogation, magnification 400×.

Hyperlipidaemia is accompanied by the increased formation of ox‐LDL 39. Studies have shown that CXCL16 is constitutively expressed in podocytes and acts as a scavenger receptor that mediates the uptake of ox‐LDL 4. According to prior studies and our preliminary experiments 40, we exposed immortalized mouse podocytes to ox‐LDL treatment for 24 hrs. We found that a great proportion of podocytes were viable (Fig. 4A), but were damaged by ox‐LDL, as the stimuli resulted in remarkable reductions in the expression of WT1, podocin and NEPH1 (Fig. 4B and C), and an up‐modulation of injury marker vimentin, compared with podocytes under normal culture conditions (*P* < 0.05; Fig. 5A). Ox‐LDL also triggered excessive expression and secretion of mmp9 (*P* < 0.05; Fig. 5B). Similar changes were confirmed by immunofluorescence (Figs 6 and 7), in which the staining of podocyte markers was weakened but injury markers were strongly stained after ox‐LDL stimulation.

**Figure 8:**
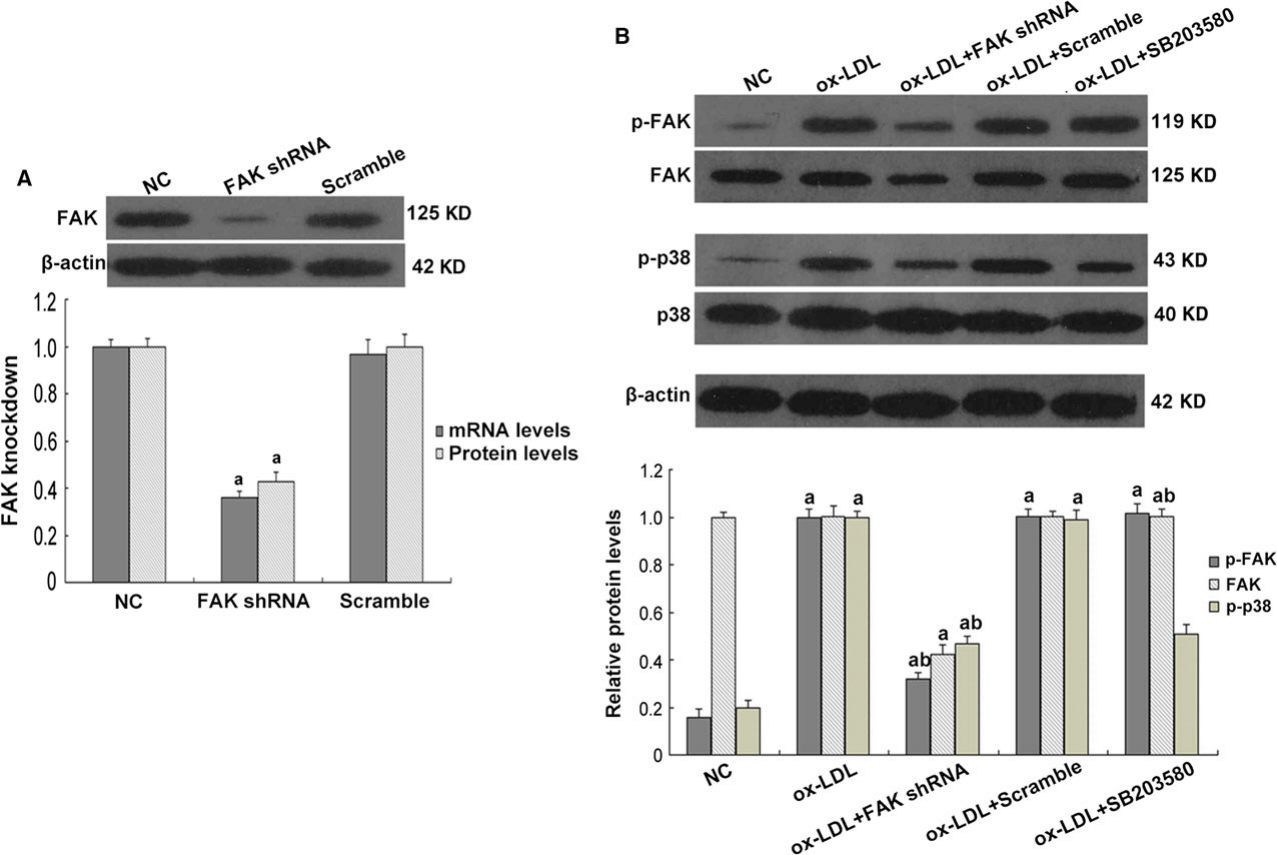
Determination of expression and activation of FAK and p38 in cultured podocytes. (**A**) Western blot and real‐time PCR results showed a significant abrogation of FAK expression on both protein and mRNA levels, presenting a relatively high knockdown efficiency of FAK gene. (**B**) A 24‐hrs incubation with ox‐LDL gave rise to FAK and p38 phosphorylation, with limited alterations on total protein expression, compared with podocytes under normal conditions. FAK expression and activation were substantially diminished by FAK gene knockdown; pre‐treatment of SB203580 dramatically deleted p38 activation, whereas showed little alterations on FAK phosphorylation or expression. Podocytes under normal conditions or transfected with scrambled shRNA were the controls. Values denote the mean ± S.D.; ^a^ *P* < 0.05 *versus* NC and ^b^ *P* < 0.05 *versus* ox‐LDL treatment.

Next, we asked whether ox‐LDL stimulation influenced FAK. We found that a 24‐hr incubation with ox‐LDL gave rise to FAK tyrosine phosphorylation, with limited alterations on total FAK expression, compared to that in podocytes under normal conditions (*P* < 0.05; Fig. 8B), which coincided with cellular changes of podocytes. To further identify the role of FAK, we constructed FAK shRNA and validated its efficiency when significant abrogation of FAK expression on both protein and mRNA levels were shown (*P* < 0.05; Fig. 8A). And ox‐LDL‐induced FAK over‐activation was substantially diminished by FAK gene knockdown (*P* < 0.05; Fig. 8B), together with the preservation of WT1 and NEPH1, and partial inhibition of injury markers vimentin and mmp9, on both protein and mRNA levels (*P* < 0.05; Figs 4B and C, 5, 6 and 7). Immunofluorescence (Figs 6 and 7) further validated the amelioration of podocyte injury, demonstrated by enhanced staining of WT1, NEPH1 and podocin, and reduced staining of vimentin and mmp9, compared with cells without FAK shRNA transfection. These observations indicated that podocytes primarily underwent adaptive mutations, which exhibited as podocyte damage, other than apoptosis when in face of ox‐LDL stimulation, during which FAK over‐activation was indispensable.

### Inhibition of FAK/p38 signalling restored podocyte injury stimulated by ox‐LDL

Focal adhesion kinase initiates a cascade of intracellular signals including c‐Src, Rho GTPase and MAPK 17, 18. P38 MAPK (p38) is a member of the MAPK family and is in close association with a variety of renal diseases 20, 21, 22. We found hyper‐activity of p38 was driven by ox‐LDL, compared with the controls (*P* < 0.05; Fig. 8B), contemporarily with the over‐activation of FAK and the mutations of cell markers (*P* < 0.05; Figs 4B and C, 5, 6, 7 and 8B). To address its potential interactions with FAK, a p38 inhibitor SB203580 was utilized. Pre‐treatment of SB203580 (5 μg/ml) for 1 hr notably obliterated ox‐LDL‐stimulated p38 activation (*P* < 0.05; Fig. 8B), with unchanged FAK expression and phosphorylation (Fig. 8B) but essentially restored cellular markers (*P* < 0.05; Figs 4B and C, 5, 6 and 7), whereas FAK gene knockdown substantially prohibited the over‐activation of both (Fig. 8B), indicating that p38 may serve as a downstream effector of FAK in response to ox‐LDL stimulation. This signalling was further identified by our *in vivo* data that TAE226 abolished the hyper‐phosphorylation of FAK and p38 in comparison with untreated HC rats (*P* < 0.05; Fig. 1C).

### Inhibition of FAK/p38 signalling reinstated ox‐LDL‐induced F‐actin re‐organization and podocyte hyper‐motility

**Figure 9:**
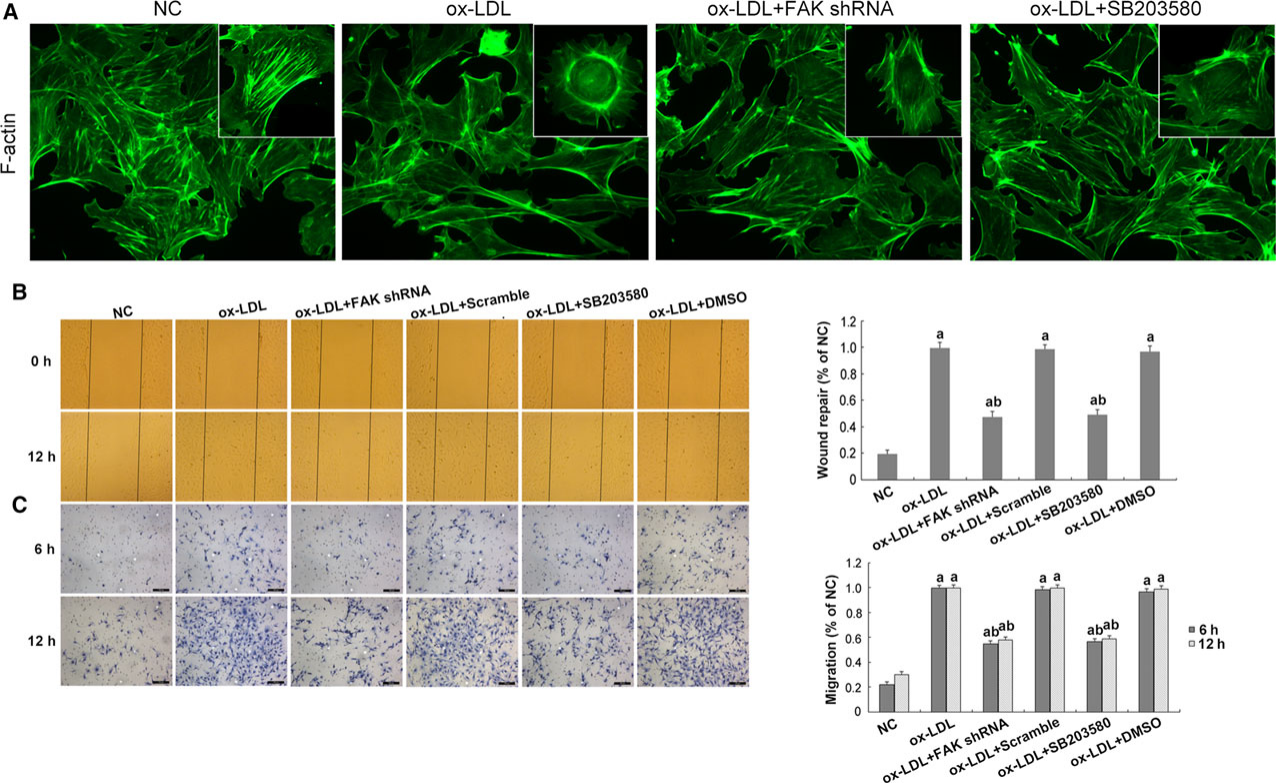
Cytoskeletal re‐organization of cultured podocytes and cell motility. (**A**) By FITC‐phalloidin staining, we observed ox‐LDL treatment for 24 hrs caused re‐organization of F‐actin, from actin bundles along the cell axis to densely interwoven filaments at the cortical regions along the cell periphery. Transfection with FAK shRNA or pre‐treatment of p38 inhibitor significantly reinstated forced F‐actin re‐assembly, demonstrated by decreased staining of F‐actin bundles at the sub‐membrane region, compared with podocytes subjected to ox‐LDL for 24 hrs. (**B**) Scratch‐wound assay was utilized to assess coordinated sheet migration of a monolayer of immortalized mouse podocytes. Podocytes treated with ox‐LDL for 12 hrs exhibited a significantly promoted increase in wound closure, compared with their counterparts; this was markedly abolished by FAK gene knockdown or SB203580 pre‐treatment. (**C**) Transwell migration assay was used to determine individual sheet migration. After ox‐LDL treatment, promoted podocyte migration was observed at as early as 6 hrs, the trend of which extended to 12 hrs of stimulation; excessive motility of podocytes were abolished by FAK shRNA transfection or p38 inhibitor pre‐treatment, where significantly diminished cell migration at either time‐point was revealed. Podocytes under normal conditions or transfected with scrambled shRNA were the controls. Values denote the mean ± S.D.; ^a^ *P* < 0.05 *versus* NC and ^b^ *P* < 0.05 *versus* ox‐LDL treatment.

Focal adhesion kinase functions as a bidirectional linkage between the actin cytoskeleton and the cell‐matrix interface 14. In this study, we found by FITC‐phalloidin staining that *in vitro* ox‐LDL treatment for 24 hrs caused F‐actin re‐organization, from actin bundles along the cell axis to densely interwoven filaments at the cortical regions along the cell periphery (Fig. 9A); transfection with FAK shRNA or pre‐treatment of SB203580 significantly reinstated forced F‐actin re‐assembly, demonstrated by decreased staining of F‐actin bundles at the sub‐membrane region, compared with podocytes subjected to ox‐LDL (Fig. 9A), implicating that ox‐LDL triggered podocyte cytoskeletal re‐organization *via* activating FAK/p38 signalling pathway.

To determine the potential impacts of F‐actin dynamics on podocyte migration under culture, wound‐healing and transwell assays were performed. Scratch‐wound assay was utilized to assess coordinated sheet migration of a monolayer of immortalized mouse podocytes. It was found that podocytes treated with ox‐LDL for 12 hrs exhibited a significantly promoted increase in wound closure, compared with their counterparts (*P* < 0.05; Fig. 9B); this was markedly prohibited by FAK gene knockdown or SB203580 pre‐treatment (*P* < 0.05; Fig. 9B). To determine individual sheet migration, podocytes pre‐treated with different incubations were seeded in the upper chamber of transwell filters and allowed to attach at 37°C for 6 and 12 hrs, respectively. After ox‐LDL treatment, promoted podocyte migration was observed at as early as 6 hrs (*P* < 0.05; Fig. 9C), the trend of which extended to 12 hrs of stimulation (*P* < 0.05; Fig. 9C). Focal adhesion kinase shRNA transfection or p38 inhibitor pre‐treatment abolished excessive motility of podocytes, where significantly diminished cell migration at either time‐point was revealed (*P* < 0.05; Fig. 9C).

In addition, we showed that ox‐LDL resulted in a substantial up‐regulation in synaptopodin expression (Fig. 7), which occurred concomitantly with increased podocyte spreading and migration but was reversed by the blockade of FAK/p38 signalling, either by FAK gene knockdown or SB203580 pre‐treatment (Fig. 7). Synaptopodin is a proline‐rich protein expressed in podocyte foot processes, responsible for podocyte actin dynamics 41. These aforementioned observations indicated that FAK/p38 signalling pathway might affect podocyte cytoskeleton *via* modulating the expression of synaptopodin.

## Discussion

Hypercholesterolaemia has long been recognized as a risk factor to the progression of kidney diseases 1. Clinical and experimental studies have provided insights into the roles of lipids and lipid‐modulating proteins as key determinants of podocyte function in health and kidney diseases 42. Joles *et al*. demonstrated in a rat model of hypercholesterolaemia that at early stage hypercholesterolaemia aggravates renal injury primarily *via* podocytes rather than other residential cells, and forced podocyte injury could secondarily lead to mesangial sclerosis 5. In the present study we showed a short‐term (6 weeks) high cholesterol diet impaired podocytes and induced mild proteinuria with a similar degree of hypercholesterolaemia; these events were associated with hyper‐activation of renal cortical FAK since TAE226 treatment or FAK inhibition demonstrated a restoration of podocyte function and proteinuria.

Focal adhesion kinase is a 125‐kD non‐receptor protein‐tyrosine kinase and has been implicated in diverse cellular events involving cell motility and proliferation *via* its auto‐phosphorylation at tyrosine 397, which thereby triggers the phosphorylation of other tyrosine residues and initiates downstream signals such as paxillin and PI3K 8, 9. Focal adhesion kinase hyper‐phosphorylation was found in several glomerular diseases such as lupus nephritis and anti‐GBM disease 10, 11. Sustained activation of FAK was also involved in ox‐LDL‐stimulated impairment of mouse macrophage migration *in vitro* 12. Hypercholesterolaemia is associated with increased formation of ox‐LDL 39. This also holds true in hypercholesterolaemia induced by a short exposure to high fat diet 43. Furthermore, there is growing evidence to suggest that the beneficial effects of statin treatment are not solely mediated by their LDL‐cholesterol lowering effect but also by additional pleiotropic effects 44. In a study in ox‐LDL‐induced human podocyte injury, de‐activation of PI3k/AKT‐signalling pathway was found and prevented by statins 40. Likewise, we showed *in vitro* ox‐LDL damaged mouse podocytes, which exhibited enhanced expression of vimentin and mmp9, a protein that was associated with ECM remodelling, when FAK and p38 were concomitantly activated by ox‐LDL treatment. Focal adhesion kinase gene interference partially reversed podocyte damage and inactivated p38, whereas p38 inhibitor did not alter FAK expression or phosphorylation, implicating that FAK mediated podocyte injury by triggering downstream effector p38 in exposure to ox‐LDL. *In vivo*, elevated p38 activity coincided with the onset of proteinuria in HC rats and could be blunted by FAK inhibition, thus it appeared that FAK/p38 axis might also be involved in renal injury under hypercholesterolaemic state.

Like mesangial cell, podocytes express scavenger receptors and can also take up lipids 4, 42. Podocyte cholesterol homeostasis is maintained by the regulation of cholesterol synthesis and intracellular trafficking 42. Lipid rafts are microdomains of the plasma membrane enriched with sphingolipids, cholesterol and protein complexes that have essential roles in signal transduction 42, 45. Cholesterol overload from its elevated carriers *i.e*. circulating LDLs, as was shown in the present study, might negatively influence the binding of podocyte SD proteins to each other, as which is assembled in lipid rafts and is playing a critical role in the formation of glomerular filtration barrier and maintenance of the interdigitating foot process pattern 42, 46, 47. We found *in vivo* and *in vitro*, SD proteins podocin and NEPH1 were dramatically decreased during the challenge of hypercholesterolaemia or ox‐LDL treatment. Focal adhesion kinase has been demonstrated as an important signalling component at focal adhesions where integrin and proteoglycan medianted adhesion links to the actin cytoskeleton 48, and also a key mediator between the actin cytoskeleton and the apical membrane of epithelial cells that contains lipid rafts 45, implying the possibility of a cytoskeletal interference system. Indeed, we illustrated the re‐organization of F‐actin stress fibres as a vital *in vitro* consequence of FAK/p38 signalling after ox‐LDL treatment, which extended from bundles along the cell axis to densely interwoven filaments at the cortical regions along the cell periphery, accompanied by enhanced cell motility. This is interesting as podocyte foot process is an actin filament‐based contractile apparatus; effacement of foot process is generally considered to represent a re‐establishment of podocyte architecture when actin filaments were replaced by a dense network of microfilament bundles 49. Our *in vivo* observations illustrated that in the absence of proteinuria, occasional focal fusion of the process occurred as a result of high cholesterol diet for 4 weeks, which developed to a diffuse involvement with concurrent proteinuria at 6 weeks, although organelles remained unchanged in both occasions. It was conceivably to believe that hypercholesterolaemia at this early stage already induced some degree of podocte injury namely focal alterations in actin cytoskeleton, which preceded the onset of proteinuria. Persistent hypercholesterolaemia was responsible for diffused effacement or further cytoskeletal re‐assembly that was associated with podocyte malfunction, as demonstrated by mutations of SD proteins and increased vimentin and mmp9 expression, and resultant proteinuria.

In addition, we surprisingly found by immunofluorescence that synaptopodin expression was increased by ox‐LDL *via* FAK/p38 signalling. Synaptopodin is a proline‐rich protein expressed in differentiated podocytes and orchestrates actin organization through its interaction with actin‐binding proteins α‐actinin‐4 and cell motility by regulating RhoA signalling; gene silencing of synaptopodin causes the impairment of cell migration 41, 50. This observation further confirmed a modulatory role of FAK/p38 in the regulation of podocyte cytoskeleton. Foot process effacement or damaged podocyte three dimensional architecture exposes podocytes to the risk of being lost by detachment as viable cells from the GBM 48; when the compensatory mechanism namely cellular hypertrophy of neighbour podocytes fails to cover the bare region of the GBM, the attachment of parietal epithelial cells to the bare GBM invariably occurs, representing a potential initial point for irreversible glomerular injury 51. Prior literature suggested that marked or long‐term hypercholesterolaemia could directly cause tubulointerstitial injury by triggering proinflammatory and profibrogenic events, which, however, was absent in short‐term hypercholesterolaemia. Therefore, early interference with podocyte injury might be a key step in the treatment of lipid disorder‐related renal diseases.

Previous studies have shown that hypercholesterolaemia‐associated podocyte stress was induced by decreased nitric oxide synthesis (NOS) and prevented by an exogenous nitric oxide donor molsidomine. It is widely accepted that nitric oxide is a potent modulator of renal blood flow and basal renal vascular tone 7. Notably, recent evidence elaborated that non‐steroidal anti‐inflammatory drugs dis‐regulated of eNOS in the kidney with a concurrent increase in levels of phosphorylated p38 52. And in another study, lipopolysaccharide‐increased macrophage motility required the participation of iNOS/Src/FAK axis 53. Thus, it would be of interest to further determine whether FAK/p38 signalling was involved in NOS activity and oxidative stress in early hypercholesterolaemia‐related podocyte injury.

Of note, in the present study no obvious alterations of the serum lipids levels were identified before or after TAE226 intervention, suggesting that TAE226 may safeguard against proteinuria *via* non‐lipid‐lowering mechanisms. Recent studies suggested in cancer cells TAE226 inhibited FAK phosphorylation with an additional effect of inhibiting insulin‐like growth factor‐I receptor (IGF‐IR) 54, whereas little information is available documenting its inhibitory effects on IGF‐IR in podocytes. If this dual effect were confirmed, developing drugs with a selective inhibition ability of FAK would be required. Alternatively, models using knockout animals would be an appropriate option for further investigations.

In summary, we showed FAK contributed to foot process effacement and proteinuria in a rat model of short‐term hypercholesterolaemia and modulated podocyte F‐actin re‐organization *via* activating p38 MAPK in response to ox‐LDL, providing evidence for early mechanisms of hypercholesterolaemia‐caused renal damage. Moreover, we presented that podocyte injury and proteinuria was prevented by FAK inhibition or TAE226, which might shed some light on developing therapeutic targets for podocytopathy.

## Conflict of interest

The authors declare no conflicts of interest.
